# Deep anterior chamber depth may be a risk factor for axial length growth in children

**DOI:** 10.3389/fmed.2024.1489989

**Published:** 2025-01-03

**Authors:** Huijie Cao, Yongguo Xiang, Hong Cheng, Kexin Sun, Shijie Zheng, Miaomiao Du, Ning Gao, Tong Zhang, Xin Yang, Jiuyi Xia, Wenjuan Wan, Ke Hu

**Affiliations:** ^1^Chongqing Key Laboratory of Ophthalmology, Chongqing Eye Institute, Chongqing Branch (Municipality Division) of National Clinical Research Center for Ocular Diseases, Department of Ophthalmology, The First Affiliated Hospital of Chongqing Medical University, Chongqing, China; ^2^Chongqing Medical University, Chongqing, China

**Keywords:** myopia, progression, axial length, anterior chamber depth, children

## Abstract

**Objective:**

To investigate and evaluate the progression of myopia and associated factors of axial length (AL) growth among children in Chongqing.

**Methods:**

This six-month prospective study was conducted on students in grades 1 to 8 at a school in Chongqing, China. All participants underwent a standard ophthalmologic examination including uncorrected visual acuity (UCVA), noncycloplegic refraction, AL, and corneal topography in March 2023. Six months later, the above examinations were repeated to obtain follow-up data. Visual habits questionnaire was gained to analyze the correlation between the AL growth and vision-related behavior.

**Results:**

A total of 417 students from Chongqing were enrolled in this study. The myopia prevalence was higher in follow-up (38.6%) than in baseline (33.3%) and the AL was longer in follow-up than in baseline (23.69 ± 1.03 mm vs. 23.57 ± 1.03 mm, *p* < 0.001). The anterior chamber depth (ACD) in students with AL growth greater than or equal to 0.2 mm (3.16 ± 0.23 mm) was deeper than that in students with AL growth less than or equal to 0.05 mm (3.02 ± 0.28 mm, *p* = 0.001), lens thickness (LT) was thinner (3.29 ± 0.10 mm vs. 3.33 ± 0.10 mm, *p* = 0.004). Furthermore, ACD was positively correlated with AL growth. (*r* = 0.181, *p* < 0.001).

**Conclusion:**

Compared to SE, AL serves as a more sensitive indicator for monitoring myopia progression. ACD was positively correlated with AL growth, and deeper ACD may be contributed to longer AL growth.

## Background

In recent years, with the prevalence of myopia increasing globally, especially in East and Southeast Asia (1), it has become a more and more serious public health concern among children. A prior study predicted that by 2050, 84% of Chinese children and teenagers aged 3 to 19 years will be affected by myopia (2). Myopia has emerged as the predominant refractive error. Furthermore, there is an escalating risk of complications associated with myopia, including myopic macular degeneration, cataracts, retinal detachment and primary open-angle glaucoma, leading to irreversible vision loss (3).

There is convincing evidence from various studies confirming that both nature (genetics and heredity) and nurture (environment and lifestyle) are serving as triggers in mediating myopia (1) (4). Epidemiological investigation have consistently demonstrated that parental myopia has contributed to be a genetic factor influencing myopia in children, with the risk of myopia showing a correlation with the number of myopic parents (5) (6). As for the environment aspects including near-vision work time, outdoors activities (7) and sleeping time (8), all of these have also been identified as potential causative factors in the development of myopia.

Referred to as axial myopia, the majority of myopic cases are linked to the excessive elongation of the eye axis (9) that affects the change of refractive status (10) (11). Unlike refractive measurement susceptible to accommodation effects, axial length (AL) measurement remains unaffected by accommodation, displaying a more stable regularity in its age-related changes. It is a crucial and objective indicator for predicting the onset and progression of myopia conveniently in children and teenagers (12). The Pentacam anterior segment analyzer, adept at capturing numerous images of the ocular anterior segment, facilitates the computation of a three-dimensional corneal map and furnishes an array of biological parameters for the anterior segment (13). As is well known, refractive errors are greatly related to the ocular biometric parameters, and they are considered to be consequences of mismatch of ocular parameters during the development. We are attempting to explore the intrinsic correlation between myopia and the parameters obtained through Pentacam.

The degree of myopia is typically assessed through the quantification of the spherical equivalent (SE) refractive error measured in diopter (1). Previous insights drawn from multiple epidemiological studies (14) (15) have established connections between the environmental factors outlined above and SE. Nevertheless, the potential association of AL with myopia-related factors and ocular parameters has not been elucidated. Our longitudinal study on school-aged adolescents aims to fill these gaps by exploring potential factors associated with the elongation of AL.

## Materials and methods

This school-based prospective study was conducted with students in grades 1 to 8 at the Fenghuang Experimental School in Shapingba District, Chongqing. The Ethics Committee of the First Affiliated Hospital of Chongqing Medical University approved the study and it was conducted in accordance with the principles of the Declaration of Helsinki. At least one parent or legal guardian of each participant was informed about the study and signed an informed consent form.

### Study population

Sampling for this study was conducted using whole cluster sampling. Briefly, two classes were randomly selected from each grade level from first grade to eighth grade and all the students in the selected classes were investigated and followed up. The exclusion criteria were as follows: (i) Suffering from severe eye diseases such as cataract, uveitis, corneal diseases and glaucoma, (ii) A diagnosis of strabismus or amblyopia, (iii) Recent history of wearing orthokeratology and (iv) Loss of follow-up or incomplete information due to various reasons. A total of 451 students were included in this school-based study.

### Methods

All participants underwent a standardized ophthalmic examination at baseline. Uncorrected visual acuity (UCVA) was assessed and recorded in LogMAR scores by using the standard logarithmic visual acuity chart at 5 meters. Noncycloplegic refraction was performed with an autorefractor (Supore, China). AL was measured with the AL-scan (NIDEK, Japan). Intraocular pressure (IOP) was assessed with a non-contact tonometer (NIDEK, Japan). Corneal and anterior chamber parameters were measured with the Pentacam AXL (Oculus Optikgeräte GmbH, Wetzlar, Germany). The quality specification section on the output graph was used to assess the quality, with an “OK” reading indicating an acceptable quality. Noncycloplegic refraction, IOP, and AL measurements were performed at the 6-month follow-up visit. All examinations were performed by skilled ophthalmologists. At least three measurements were made for each eye and averaged, but only the right eye of each participant was selected for statistical analysis. Spherical equivalent (SE) equals diopter (D) of spherical power plus 1/2 diopter (D) of cylindrical power. Myopia was defined as SE ≤ −0.5D and UCVA >0 logMAR. In addition, to investigate the association between lifestyle and the progression of myopia, students were required to complete questionnaires about genetic and environmental issues that may contribute to myopia at the 6-month follow-up visit. The questionnaire included information on average daily outdoor activity, digital screens use, and sleep time over the past 6 months. The questionnaires were completed by the students themselves or with the help of their parents.

The investigated indices were as follows: UCVA, SE, AL, IOP, elongation of the AL (ΔAL = AL at 6-month follow up - AL at baseline), AL to corneal radius of curvature ratio (AL / CR), mean curvature power of the cornea within the central 3-mm circle (Km), astigmatism of the front surface (Astig. F), astigmatism of the back surface (Astig. B), corneal thickness at the apex (CCT), white to white distance (WTW), internal anterior chamber depth (ACD), mean anterior chamber angle (ACA), anterior chamber volume at 10 mm diameter (ACV), eccentricity of the front surface of the cornea (ECC. F), lens thickness (LT), and eccentricity of the back surface of the cornea (ECC. B).

### Statistical analysis

The statistical analysis was performed with SPSS version 26 (IBM Corp. in Armonk, NY, USA). One-sample Kolmogorov–Smirnov tests and the normal distribution histogram method were used to assess the normality of the distributions of the continuous variables. Paired samples *t*-test was employed to compare normally distributed continuous variables, and Mann–Whitney *U* test was used to compare skewed continuous variables. The chi-squared test was utilized to compare categorical variables. Pearson’s correlation analysis was used to analyze the correlation between ΔAL and ocular parameters. Stepwise multiple linear regression analysis was conducted to explore the relationship between ΔAL and ocular parameters. The significance level was set at *p* < 0.05.

## Results

### General information

At the 6-month follow-up, 34 students were excluded for wearing corneal contact lenses or transferring to another school, resulting in a total of 417 students, 202 (48.4%) boys and 215 (51.6%) girls, aged between 7 and 14 years, participating in the study. The number of students in each grade group is equal approximately and the gender ratio is close to 1:1 (Table 1).

**Table 1:** Distribution of participants by grade level.

Grade	Cases	Male	Age (year)
1	55	26 (47.2%)	7.7 ± 0.4
2	49	21 (42.8%)	8.7 ± 0.3
3	45	21 (46.6%)	9.7 ± 0.3
4	43	20 (46.5%)	10.7 ± 0.4
5	52	30 (57.6%)	11.8 ± 0.3
6	44	19 (43.1%)	12.7 ± 0.4
7	61	24 (39.3%)	13.7 ± 0.4
8	68	41 (60.2%)	14.8 ± 0.4
Total	417	202 (48.4%)	11.4 ± 2.5

### Myopia progression

**Figure 1:**
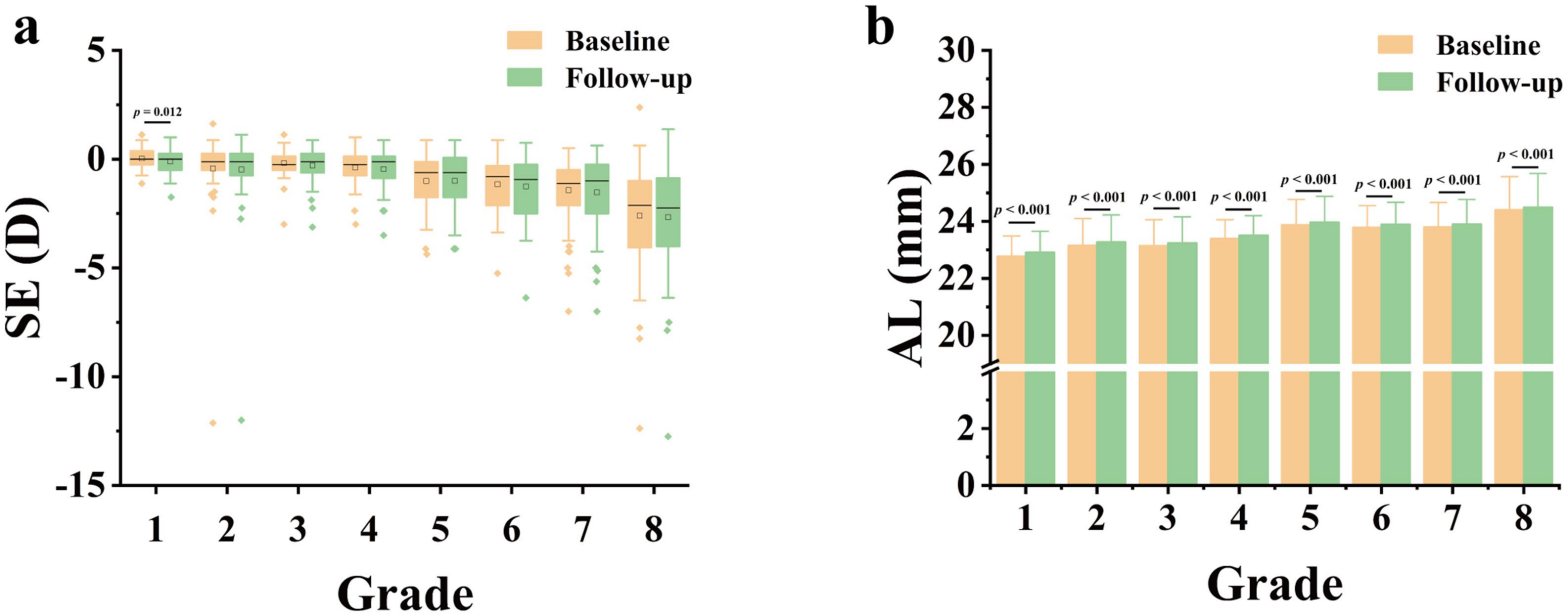
Comparison of spherical equivalent (SE, A**)** and axial length (AL, B**)** at 6-month follow-up versus AL and SE at baseline. **D**, diopter.

As displayed in Table 2, the myopia rate increased in all grade groups expect in 4th grade, and the overall myopia prevalence in baseline was 33.3%, which was increased by 5.3% as compared to that in follow-up (38.6%), with no statistically significant difference (*p* = 0.112). Apart from 1st grade, no significant differences in the change of SE were observed among each grade groups (Figure 1A). However, the median (quartile) SE progression for this sample set was lower in follow-up [−0.50 (−1.63, 0) D] than in baseline [−0.50 (−1.50, 0) D, *p* < 0.05]. As for AL, there was a significant increase in all grade groups (Figure 1B), and the average AL had significantly higher in follow-up than in baseline (23.69 ± 1.03 mm vs. 23.57 ± 1.03 mm, *p* < 0.001).

**Table 2:** Comparison of myopia progress in different grades.

	Grade	Cases	Baseline	Follow-up	*p*
Myopia prevalence (%)	1	55	3.6	10.9	0.142
	2	49	8.2	16.3	0.218
	3	45	8.9	13.3	0.502
	4	43	20.9	20.9	1.000
	5	52	42.3	44.2	0.843
	6	44	47.7	56.8	0.393
	7	61	47.5	55.7	0.365
	8	68	70.6	73.5	0.702
	Total	417	33.3	38.6	0.112
SE [D, Median (P25, P75)]	1	55	0 (−0.25, 0.38)	0 (−0.50, 0.25)	0.012
	2	49	−0.13 (−0.50, 0.25)	−0.13 (−0.81, 0.25)	0.357
	3	45	−0.25 (−0.56, 0.19)	−0.13 (−0.63, 0.25)	0.322
	4	43	−0.25 (−0.75, 0.13)	−0.13 (−0.88, 0.13)	0.569
	5	52	−0.63 (−1.81, −0.13)	−0.63 (−1.81, 0.09)	0.869
	6	44	−0.81 (−2.13, −0.28)	−0.94 (−2.56, −0.19)	0.088
	7	61	−1.13 (−2.13, −0.44)	−1.00 (−2.56, −0.19)	0.116
	8	68	−2.13 (−4.09, −1.00)	−2.25 (−4.00, −0.88)	0.374
	Total	417	−0.50 (−1.50, 0)	−0.50 (−1.63, 0)	0.002
AL (mm, Mean ± SD)	1	55	22.77 ± 0.72	22.91 ± 0.74	< 0.001
	2	49	23.15 ± 0.95	23.27 ± 0.96	< 0.001
	3	45	23.14 ± 0.92	23.24 ± 0.92	< 0.001
	4	43	23.39 ± 0.67	23.51 ± 0.69	< 0.001
	5	52	23.87 ± 0.90	23.96 ± 0.92	< 0.001
	6	44	23.78 ± 0.77	23.89 ± 0.78	< 0.001
	7	61	23.80 ± 0.86	23.90 ± 0.87	< 0.001
	8	68	24.40 ± 1.17	24.49 ± 1.19	< 0.001
	Total	417	23.57 ± 1.03	23.69 ± 1.03	< 0.001

SE, spherical equivalent; D, diopter; AL, axial length. Myopia was defined as SE ≤ −0.5D and UCVA > 0 logMAR.

According to ΔAL, we divided students into two groups, with ΔAL greater than or equal to 0.2 mm for Group A and smaller than or equal to 0.05 mm for Group B. A total of 60 and 117 students were included in Group A and Group B, respectively. We compared the eye parameters of different growth rate groups of AL. As illustrated in Table 3, the SE at baseline was lower (*p* = 0.006), No. of myopic parents was greater (*p* < 0.001), and time spent outdoors was shorter (*p* = 0.001) in Group A than in Group B. In addition, ACD in Group A (3.16 ± 0.23 mm) was deeper than that in Group B (3.02 ± 0.28 mm, *p* = 0.001), LT in Group A (3.29 ± 0.10 mm) was thinner than that in Group B (3.33 ± 0.10 mm, *p* = 0.004), and this difference persisted after correcting for the number of myopic parents and time spent outdoors (*p* = 0.017).

**Table 3:** Comparison of ocular parameters between different growth rate groups in AL.

	Group A *n* = 60	Group B *n* = 117	*P*
Sex (Male / %)	29 / 48.3%	59 / 50.4%	0.792
Age (year)	11 (8.9, 13.48)	11.9 (10, 14.05)	0.089
SE (D)	−0.88 (−2.09, −0.25)	−0.38 (−1.13, 0.13)	0.006
AL (mm)	23.56 (22.97, 24.19)	23.54 (23.09, 24)	0.825
Δ SE (D)	−0.19 (−0.5, 0)	0.13 (−0.13, 0.25)	< 0.001
Δ AL (mm)	0.28 (0.22, 0.34)	0.02 (−0.01, 0.03)	< 0.001
IOP (mmHg)	17 (15, 19)	16 (15, 19)	0.158
Km (D)	43.5 (42.4, 44.9)	42.9 (42.15, 43.95)	0.013
Asitg.F (D)	1.1 (0.73, 1.4)	1.1 (0.7, 1.5)	0.938
CCT (μm)	547.43 ± 33.4	550.10 ± 29.3	0.585
ACD (mm)	3.16 ± 0.23	3.02 ± 0.28	0.001
ACA (degree)	41.64 ± 3.50	40.41 ± 4.72	0.053
ACV (μL)	181.17 ± 28.19	169.79 ± 29.26	0.014
LT (mm)	3.29 ± 0.10	3.33 ± 0.10	0.004
WTW (mm)	11.01 ± 2.84	11.29 ± 2.05	0.443
ECC.F	0.57 (0.46, 0.66)	0.55 (0.49, 0.62)	0.436
ECC.B	0.19 (−0.01, 0.31)	0.2 (0.02, 0.31)	0.835
No. of myopic parents	1 (0, 1)	0 (0, 1)	< 0.001
Time spent on digital screens (h)	2 (1, 3)	2 (1, 2)	0.235
Time spent outdoors (h)	2 (1, 4)	3 (2, 4)	0.001
Sleeping time (h)	9 (8, 9.75)	9 (8, 9)	0.609

Group A: students with ΔAL greater than or equal to 0.2 mm. Group B: students with ΔAL smaller than or equal to 0.05 mm.

SE, spherical equivalent; D, diopter; AL, axial length; IOP, intraocular pressure; ΔSE: SE at follow up - SE at baseline; ΔAL: AL at follow up - AL at baseline; Km, mean curvature power of the cornea within the central 3-mm circle; Astig. F, astigmatism of the front surface; CCT, corneal thickness at the apex; ACD, internal anterior chamber depth; ACA, mean anterior chamber angle; ACV, anterior chamber volume at 10 mm diameter; LT, lens thickness; WTW, white to white distance; ECC.F, eccentricity of the front corneal surface, ECC.B, eccentricity of the back corneal surface.

### Correlation analysis

Pearson’s correlation analysis was performed to analyze the correlation of the AL growth with ocular parameters and myopia related factors. As shown in Table 4, Km (*r* = 0.100, *p* = 0.042), ACD (*r* = 0.181, *p* < 0.001), ACA (*r* = 0.116, *p* = 0.017), ACV (*r* = 0.128, *p* = 0.009), No. of myopic parents (*r* = 0.278, *p* < 0.001), and time spent on digital screens (*r* = 0.123, *p* = 0.012) were positively correlated with the growth of AL. While age (*r* = −0.115, *p =* 0.019), LT (*r* = −0.174, *p* < 0.001) and time spent outdoors (*r* = −0.212, *p* < 0 0.001) were negatively correlated with the growth of AL. Nevertheless, there was no statistically significant correlation between the growth of AL and IOP (*r* = 0.005, *p* = 0.926), nor the sleeping time (*r* = −0.010, *p* = 0.843).

**Table 4:** Correlation analysis of ocular parameters with the growth of the axial length.

Ocular parameters	Correlation coefficient	*p*
Age	−0.115	0.019
IOP	0.005	0.926
Km	0.100	0.042
Asitg. F	−0.013	0.791
CCT	−0.043	0.377
ACD	0.181	< 0.001
ACA	0.116	0.017
ACV	0.128	0.009
LT	−0.174	< 0.001
WTW	0.071	0.158
ECC. F	−0.035	0.474
ECC. B	−0.019	0.706
No. of myopic parents	0.278	< 0.001
Time spent on digital screens	0.123	0.012
Time spent outdoors	−0.212	< 0.001
Sleeping time	−0.010	0.843

Pearson’s correlation analysis.

IOP, intraocular pressure; Km, mean curvature power of the cornea within the central 3-mm circle; Astig. *F*, astigmatism of the front surface; CCT, corneal thickness at the apex; ACD, internal anterior chamber depth; ACA, mean anterior chamber angle; ACV, anterior chamber volume at 10 mm diameter; LT, lens thickness; WTW, white to white distance; ECC.F, eccentricity of the front corneal surface, ECC.B, eccentricity of the back corneal surface.

### Stepwise multiple linear regression

Stepwise multiple linear regression analysis was applied to further explore the relationship between the growth of AL and factors mentioned above. Table 5 displayed that only the number of myopic parents, time spent outdoors, LT, ACD, age and time spent on digital screens were retained in the final model (*R*^2^ = 0.163). These factors explained 16.3% of the growth of AL.

**Table 5:** Associations between the growth of the axial length and possible risk factors.

	Δ AL		
	*B*	Sta.β	*p*
No. of myopic parents	0.032	0.219	< 0.001
Time spent outdoors (h)	−0.012	−0.149	0.001
LT (mm)	−0.123	−1.21	0.018
Age (year)	−0.007	−0.180	< 0.001
ACD (mm)	0.052	0.144	0.007
Time spent on digital screens (h)	0.011	0.124	0.008
Adjusted *R*^2^	0.163		

Multiple stepwise linear regression.

Δ AL, axial length (AL) at 6-month follow-up - AL at baseline; *B*, Unstandardized Coefficient B, Sta.β, Standardized Coefficient Beta; ACD, internal anterior chamber depth; LT, lens thickness; h, hour.

## Discussion

Myopia swept across the world over the past three decades, it has become a serious public health problem necessitating urgent solutions. Our longitudinal investigation of school-aged students over a six-month period detected a rise in myopia prevalence and AL. Notably, ACD exhibited a positive correlation with AL growth, suggesting that deeper ACD may be contributed to longer AL growth.

Our study meticulously compared data on changes in myopia prevalence, SE and AL between the baseline and the follow-up. We observed that not only did the total myopia prevalence increase, but there was also a slight uptick in almost all grade groups after 6 months. The median (quartile) SE progression for all grade group excluding 1^st^ grade was lower in the follow-up than that in the baseline. A significant rise in AL was evident across all grade groups, with the average AL notably longer in the follow-up compared to the baseline. These findings align with prior researches (1, 16), showing school-aged children are prone to myopia. It may stem from the inherently unstable ocular conditions of during adolescence, a pivotal developmental phase, susceptible to the influences of genetic and environmental factors. Interestingly, our study revealed an overall SE progression of 0 (−0.25, 0.25) D, indicating a slower pace compared to other surveys reporting myopia progression of −0.3D in 6 months or − 0.6D in a year (17, 18). This result is consistent with the former longitudinal studies conducted in North America (18–20), demonstrating that myopia progression showed a seasonal correlation and eye growth was slower in the summer. Our six-month research, spanning from March to September, inclusive of the entire summer, benefited from ample ambient illuminance, potentially mediating protective effects against myopia through retinal dopamine release (21). Another possible reason is that the school we investigated was located in an area close to rural areas and the students had less academic pressure compared their urban counterparts. Their lower progression of myopia showed regional disparity consistent with previous studies (22).

It is noteworthy that statistical significance was observed only in the overall sample for myopia prevalence and SE progression were statistically significant, not for each grade group. We attribute this to the modest progression degree and marginally smaller sample size in individual grade groups, leading to insufficient significance in the progression while the total sample size is sufficient, thus exhibiting statistical differences. Nevertheless, changes in AL demonstrated statistical significance not only in the entire sample, but also within each grade group. AL increases correspondingly during ocular development and is closely linked to SE changes (10, 11). Compared to SE, subtle differences of AL in small sample sizes were detectable and statistically significant, indicating that AL may emerge as a more sensitive indicator than SE to monitor myopia occurrence and progression. In our study, the SE was measured by noncycloplegic refraction, ensuring high accuracy in adolescent myopia screening and monitoring. However, it cannot fully replace cycloplegic refraction currently, as it is influenced by ocular accommodation (23). In contrast, AL measurements were obtained through optical biometry, a non-contact method with advantages of minimal measurement error, convenience and speed (24). Compared to computer optometry in myopia screening, AL demonstrates high accuracy and does not necessitate medication for ciliary muscle paralysis (25). This not only streamlines the examination process and saves time, but also avoids adverse reactions caused by ciliary muscle paralysis in students. Building on these advantages, our study provides additional evidence supporting AL as a more precise tool for monitoring myopia progression than SE. It holds the potential for widespread application in the screening and monitoring of myopia in public health for children, thus augmenting the effectiveness of myopia prevention and control.

We categorized the students into two groups based on ΔAL and conducted a comparative analysis of ocular parameters between the two groups. The findings revealed that students exhibiting greater AL increments tended to have deeper ACD and thinner LT. Given the notable growth of AL, we have performed Pearson’s correlation analysis to analyze AL growth with ocular parameters and myopia related factors. The results suggested that ACD mentioned above showed a statistically significant positive correlation with ΔAL. AL encompasses corneal central thickness (CCT), ACD, LT, and vitreous cavity depth (VCD) (10), each of which influences the refractive state of eyes. A previous study analyzing biometric parameters across different degrees of myopia found that patients with moderate myopia experienced an increase in ACD and a decrease in LT compared to those with low myopia (26). In a prospective cohort study on retinopathy of prematurity (ROP), eyes affected by ROP exhibited shallower ACD, higher LT, and greater degrees of refractive errors with similar AL (27). Shih et al. revealed that AL and ACD increased with the severity of myopia (28). Lee et al. found the ACD of myopia in children is the deepest, and that of hyperopia is the shallowest (29). In our research, we found similar results as before. Currently, there are few reports on the association between ACD and myopia. During eyeball development, as axial length increases, the refractive state undergoes a gradual shift from hyperopia to emmetropia, eventually progressing to myopia. The hyperopia state preceding emmetropia is termed hyperopia reserve (HR) (expressed as spherical equivalent, SE). Children experience smaller SE changes with the same AL increment compared to adults. We hypothesize that the deepening of ACD is offset by the decrease in refractive index resulting from lens thinning. Due to the decrease in refractive index of lens, visual imaging is located behind the retina. Prolonged exposure to this stimulus leads to a trend of AL growth. Consequently, children with deeper ACD exhibited thinner LT and higher AL growth, as evident in our results. Additionally, another point is that axial myopia may primarily arise from abnormal changes in the anterior segment structure. In light of these findings, deep ACD may be a risk factor for AL growth in children, demonstrating the ability to predict progression in the early stages of myopia like AL does. Even when SE fails to exhibit significant changes due to hyperopia reserve, both AL and ACD have undergone variations and can be detected. Our results on ACD contribute to an additional perspective for identifying myopia risk through ACD.

Moreover, evidence supports that children with myopic parents are more likely to develop myopia compared to those with non-myopic parents and outdoor-activities have a protective effect on the development and progression of myopia (30). This protective effect may be attributed to the heightened exposure to natural light outdoors, a notion reinforced by animal research findings in chicks, strongly indicating that light intensity influences myopia development (31). These findings align with our study. Moreover, we found a significant correlation between time spent digital screens and ΔAL that in line with prior research (32). Intriguingly, we observed a negative correlation between age and ΔAL. Studies have shown that during the normal development of human eyes, AL increases rapidly in infancy and young children, with the growth rate gradually slowing down and stabilizing around the age of 13 (33–35). One plausible explanation could be the gradual cessation of eye development with age. Additionally, the change in diopter caused by AL growth can be compensated by the lens refractive power without significant alteration, establishing a certain safe allowable range for AL growth. Considering the negative correlation between lens refractive power and age (36), the safe allowable range for AL growth diminishes with age. Our findings aim to contribute valuable evidence for formulating a safe allowable range for AL growth.

Subsequently, we employed multiple stepwise linear regression analysis to delve deeper into the relationship between ΔAL and the aforementioned factors. In the final model, only the number of myopic parents, outdoor activity time, LT, ACD, age, and time spent on digital screens were retained. According to the standardized regression coefficients, we found that LT, the number of myopic parents, age and ACD wielded a more substantial impact on ΔAL, followed by time spent outdoors and time spent on digital screens. However, the model exhibited suboptimal fit and the explanatory power of it was slightly insufficient which can explain only 16.3% of ΔAL. We speculate that there may exist additional influencing factors not yet incorporated into this model. While we primarily relied on the precedents set by researchers to include the influencing factors of myopia for testing, we acknowledge the challenge of identifying all independent variables. Additionally, the modest explanatory power could be attributed to the relatively small sample size.

However, this study has several limitations. Firstly, the short time span and the relatively small sample size of each grade group results in insignificant statistical differences. Even though the sample size was small, we still conducted detailed visual acuity, optometry, AL and anterior ocular parameters examinations to analyze. Therefore, we will further expand the sample size to conduct prospective studies and verify our conclusions. Secondly, the noncycloplegic refraction results cannot completely replace cycloplegic refraction, but we had tried to obtain the true optometry through repeating measurements. Thirdly, the data on time spent on digital screens, time spent outdoors and sleeping time were not measured directly but were obtained through a questionnaire, which may have contributed to recall bias. To minimize these bias, questionnaire should be completed by both the children and their parents.

In conclusion, this survey gathered data from school-age children in order to track myopia progression, scrutinize AL growth and explore associated influencing factors. Our findings propose that AL stands out as a more sensitive indicator compared to SE for monitoring myopia progression. Notably, deep ACD may be a risk factor for AL growth. The measurement of ACD introduces novel perspectives for predicting AL growth and evaluating the efficacy of existing measures for myopia prevention and control.

## Data Availability

The datasets generated and/or analyzed in this article are available from the corresponding author upon reasonable request (KH, cqhuke@hospital.cqmu.edu.cn).

## References

[1] Baird PN Saw SM Lanca C Guggenheim JA Smith III EL Zhou X. Myopia. Nat Rev Dis Primers. (2020) 6:99. doi: 10.1038/s41572-020-00231-4 33328468

[2] Dong L Kang YK Li Y Wei WB Jonas JB. Prevalence and Time Trends of Myopia in Children and Adolescents in China: a systemic review and meta-analysis. Retina. (2020) 40:399–411. doi: 10.1097/IAE.0000000000002590 31259808

[3] Chiang SY Weng TH Lin CM Lin SM. Ethnic disparity in prevalence and associated risk factors of myopia in adolescents. J Formos Med Assoc. (2020) 119:134–43. doi: 10.1016/j.jfma.2019.03.004, PMID: 30910275

[4] Wang J Li Y Musch DC Wei N Qi X Ding G. Progression of myopia in school-aged children after COVID-19 home confinement. JAMA Ophthalmol. (2021) 139:293–300. doi: 10.1001/jamaophthalmol.2020.6239, PMID: 33443542 PMC7809617

[5] Mutti DO Mitchell GL Moeschberger ML Jones LA Zadnik K. Parental myopia, near work, school achievement, and children’s refractive error. Invest Ophth Vis Sci. (2002) 43:3633–40. 12454029

[6] You QS Wu LJ Duan JL Luo YX Liu LJ Li X. Factors associated with myopia in school children in China: the Beijing childhood eye study. PLoS One. (2012) 7:e52668. doi: 10.1371/journal.pone.0052668 23300738 PMC3531363

[7] Wu PC Chen CT Lin KK Sun CC Kuo CN Huang HM. Myopia prevention and outdoor light intensity in a school-based cluster randomized trial. Ophthalmology. (2018) 125:1239–50. doi: 10.1016/j.ophtha.2017.12.011, PMID: 29371008

[8] Jee D Morgan IG Kim EC. Inverse relationship between sleep duration and myopia. Acta Ophthalmol. (2016) 94:e204–10. doi: 10.1111/aos.12776, PMID: 26031352

[9] Young TL. Complex trait genetics of refractive error. Arch Ophthalmol. (2007) 125:38. doi: 10.1001/archopht.125.1.38 17210850

[10] Richter GM Wang M Jiang X Wu S Wang D Torres M. Ocular determinants of refractive error and its age- and sex-related variations in the Chinese American eye study. JAMA Ophthalmol. (2017) 135:724–32. doi: 10.1001/jamaophthalmol.2017.1176, PMID: 28520882 PMC5710201

[11] Mutti DO Hayes JR Mitchell GL Jones LA Moeschberger ML Cotter SA. Refractive error, axial length, and relative peripheral refractive error before and after the onset of myopia. Investig Opthalmology Vis Sci. (2007) 48:2510–9. doi: 10.1167/iovs.06-0562, PMID: 17525178 PMC2657719

[12] Tao L Wang C Peng Y Xu M Wan M Lou J. Correlation between increase of axial length and height growth in Chinese school-age children. Front Public Health. (2022) 9:817882. doi: 10.3389/fpubh.2021.817882, PMID: 35127628 PMC8811027

[13] Xu Y Ye Y Xian Y Niu L Zhou X Zhao J. Comparison of corneal and lens density measurements obtained by Pentacam and CASIA2 in myopes. BMC Ophthalmol. (2023) 23:448. doi: 10.1186/s12886-023-03199-3, PMID: 37950259 PMC10636911

[14] He M Xiang F Zeng Y Mai J Chen Q Zhang J. Effect of time spent outdoors at school on the development of myopia among children in China: a randomized clinical trial. JAMA. (2015) 314:1142–8. doi: 10.1001/jama.2015.10803, PMID: 26372583

[15] Tao ZY Chen SQ Tang Y Zhao J Wang J Lin ZH. The influence of parents’ background and their perception on the progression of myopia in children. Int J Clin Pract. (2022) 2022:1–7. doi: 10.1155/2022/4123470 PMC10292946 37377847

[16] Donovan L Sankaridurg P Ho A Chen X Lin Z Thomas V. Myopia progression in Chinese children is slower in summer than in winter. Optom Vis Sci. (2012) 89:1196–202. doi: 10.1097/OPX.0b013e3182640996, PMID: 22797511 PMC4696401

[17] Chen Y Drobe B Zhang C Singh N Spiegel DP Chen H. Accommodation is unrelated to myopia progression in Chinese myopic children. Sci Rep. (2020) 10:12056. doi: 10.1038/s41598-020-68859-6 32694658 PMC7374687

[18] Fujiwara M Hasebe S Nakanishi R Tanigawa K Ohtsuki H. Seasonal variation in myopia progression and axial elongation: an evaluation of Japanese children participating in a myopia control trial. Jpn J Ophthalmol. (2012) 56:401–6. doi: 10.1007/s10384-012-0148-1, PMID: 22669350

[19] Fulk GW Cyert LA. Can bifocals slow myopia progression? J Am Optom Assoc. (1996) 67:749–54. PMID: 9286316

[20] Goss DA Rainey BB. Relation of childhood myopia progression rates to time of year. J Am Optom Assoc. (1998) 69:262–6. PMID: 9585666

[21] Feldkaemper M Schaeffel F. An updated view on the role of dopamine in myopia. Exp Eye Res. (2013) 114:106–19. doi: 10.1016/j.exer.2013.02.007, PMID: 23434455

[22] Saxena R Vashist P Tandon R Pandey RM Bhardawaj A Menon V. Prevalence of myopia and its risk factors in urban school children in Delhi: the North India myopia study (NIM study). PLoS One. (2015) 10:e0117349. doi: 10.1371/journal.pone.0117349 25719391 PMC4342249

[23] Guo X Shakarchi AF Block SS Friedman DS Repka MX Collins ME. Noncycloplegic compared with cycloplegic refraction in a Chicago school-aged population. Ophthalmology. (2022) 129:813–20. doi: 10.1016/j.ophtha.2022.02.027, PMID: 35245603

[24] Sankaridurg P Tahhan N Kandel H Naduvilath T Zou H Frick KD. IMI impact of myopia. Investig Opthalmol Vis Sci. (2021) 62:2. doi: 10.1167/iovs.62.5.2, PMID: 33909036 PMC8083082

[25] Xiong S Lv M Zou H Zhu J Lu L Zhang B. Comparison of refractive measures of three autorefractors in children and adolescents. Optom Vis Sci. (2017) 94:894–902. doi: 10.1097/OPX.0000000000001113, PMID: 28816868 PMC5571878

[26] Neroev VV Tarutta EP Khanjian AT Harutyunyan SG Markosian GA Khodzhabekyan NV. Optical aberrations of the eyes with various degrees of myopia. Vestn oftal’mologii. (2021) 137:14. doi: 10.17116/oftalma202113705114 34726853

[27] Lee YS Chang SHL Wu SC See LC Chang SH Yang ML. The inner retinal structures of the eyes of children with a history of retinopathy of prematurity. Eye. (2018) 32:104–12. doi: 10.1038/eye.2017.156, PMID: 28776594 PMC5770707

[28] Shih YF Chen TC Chiang TH Lin LLK Hung PT. Changes of anterior segment during childhood: a biometric study. J Med Ultrasound. (2011) 19:33–40. doi: 10.1016/j.jmu.2011.05.004

[29] Lee JW Yau GS Woo TT Yick DW Tam VT Yuen CY. The anterior chamber depth and retinal nerve fiber layer thickness in children. Sci World J. (2014) 2014:538283:1–5. doi: 10.1155/2014/538283 PMC4241318 25431789

[30] Goldschmidt E Jacobsen N. Genetic and environmental effects on myopia development and progression. Eye. (2014) 28:126–33. doi: 10.1038/eye.2013.254, PMID: 24357837 PMC3930266

[31] Ashby RS Schaeffel F. The effect of bright light on lens compensation in chicks. Investig Opthalmol Vis Sci. (2010) 51:5247–53. doi: 10.1167/iovs.09-4689, PMID: 20445123

[32] Lin LLK Shih YF Hsiao CK Chen CJ. Prevalence of myopia in Taiwanese schoolchildren: 1983 to 2000. Ann Acad Med Singap. (2004) 33:27–33. doi: 10.47102/annals-acadmedsg.V33N1p27 15008558

[33] Rozema JJ Herscovici Z Snir M Axer-Siegel R. Analysing the ocular biometry of new-born infants. Ophthalmic Physiol Opt. (2018) 38:119–28. doi: 10.1111/opo.12433, PMID: 29285779

[34] Axer-Siegel R Herscovici Z Davidson S Linder N Sherf I Snir M. Early structural status of the eyes of healthy term neonates conceived by in vitro fertilization or conceived naturally. Invest Ophthalmol Vis Sci. (2007) 48:5454. doi: 10.1167/iovs.07-0929 18055792

[35] Chen S Guo Y Han X Yu X Chen Q Wang D. Axial growth driven by physical development and myopia among children: a two year cohort study. J Clin Med. (2022) 11:3642. doi: 10.3390/jcm11133642, PMID: 35806925 PMC9267224

[36] Xiong S Zhang B Hong Y He X Zhu J Zou H. The associations of lens power with age and axial length in healthy Chinese children and adolescents aged 6 to 18 years. Investig Opthalmology Vis Sci. (2017) 58:5849–55. doi: 10.1167/iovs.17-22639, PMID: 29141080

